# Novel 12 Mb interstitial deletion of chromosome 8p11.22-p21.2: a case report

**DOI:** 10.1186/s12920-022-01274-0

**Published:** 2022-06-06

**Authors:** Jincheng Dai, Jun Zeng, Hongxi Tan, Xiangsheng Cai, Benqing Wu

**Affiliations:** 1 Department of Paediatrics, University of Chinese Academy of Sciences-Shenzhen Hospital, Jinan University, Guangzhou, China; 2 Center for Medical Experiments, University of Chinese Academy of Sciences-Shenzhen Hospital, Shenzhen, China

**Keywords:** Chromosome deletion, 8p11.22-p21.2, Kallmann syndrome, *FGFR1*, Case report

## Abstract

**Background:**

The deletion of a short arm fragment on chromosome 8 is a rare cause of Kallmann syndrome and spherocytosis due to deletion of the *FGFR1* and *ANK1* genes.

**Case presentation:**

This case study describes a 4-month-old child with growth and psychomotor retardation, auricle deformity, microcephaly, polydactyly, a heart abnormality, and feeding difficulties. An approximately 12.00 MB deletion was detected in the 8p11.22-p21.2 region of chromosome 8. After sequencing, we found that 65 protein genes had been deleted, including *FGFR1*, which resulted in Kallmann syndrome. There was no deletion of the *ANK1* gene associated with spherocytosis, consistent with the phenotype.

**Conclusion:**

This patient is a new case of short arm deletion of chromosome 8, resulting in novel and previously unreported clinical features.

## Background

Short arm deletions of chromosome 8 in people are rare. Since the first report of a short arm deletion of chromosome 8 related to spherocytosis [1], ten additional cases have been reported globally. White et al. [2] noted that deletion of the *ANK1* gene on chromosome 8 was associated with spherocytosis. Dodé et al. [3] reported that deletion of the *FGFR1* gene resulted in Kallmann syndrome. Mu et al. [4] described a more extensive range of heterozygous deletions on chromosome 8, including the SLC20A2 and THAP1 genes, which led to dystonia. Tham et al. [5] proposed that deletion of KAT6A resulted in a syndrome that included heart defects, intellectual disability, difficulty eating, craniocerebral protrusion, and unique facial features. This case study reports a new 12 MB deletion in the short arm 8p11.22-p21.2 of chromosome 8.

## Case presentation

**Fig. 1:**
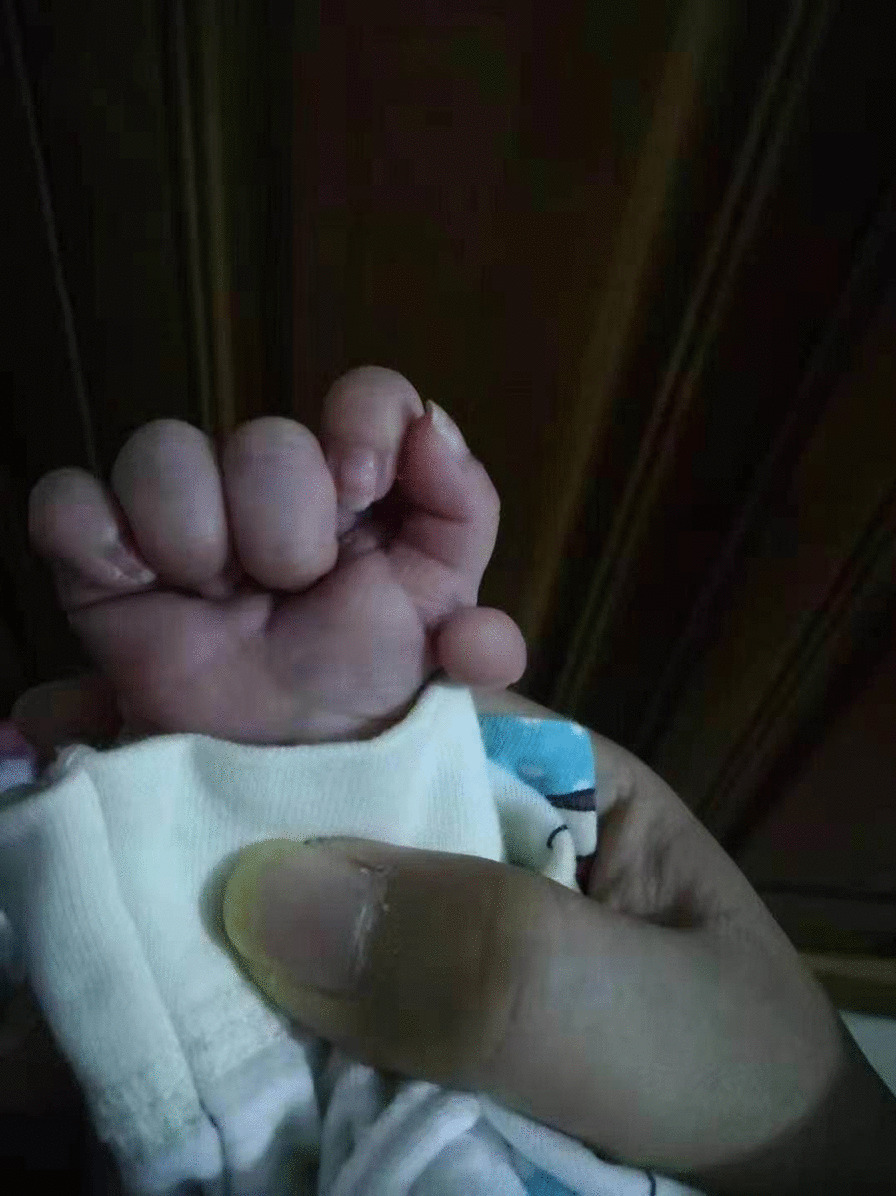
The patient has six congenital fingers

A 4-month, 20-day-old girl was admitted to the University of Chinese Academy of Science-Shenzhen Hospital on February 8, 2021, with a cough that had lasted six days and a fever for three h. She was a full-term child whose parents were not close relatives. There was no history of radiation exposure, smoking, or drinking during pregnancy. Additionally, there was no history of cold, fever, medication, nontoxic substances, or radiation exposure during early pregnancy. The child was the fourth fetus and second birth in the family. She was delivered vaginally at a gestational age of 39 + 3 weeks with a birth weight of 2.7 kg. During delivery, there was no asphyxia, umbilical cord around the neck, or premature rupture of membranes; the amniotic fluid was clear, and the placenta was normal. Her Apgar scores 1 and 5 min after birth were both 10. She had six fingers on her right hand (Fig. 1). Two days after birth, after feeding poorly, moaning for half a day, and developing a fever for 1 h, she was diagnosed with neonatal purulent meningitis, sepsis, pneumonia, and hyperbilirubinemia. Nineteen days after birth, a swollen, dark tumor developed on the child's right thumb. Due to evident ischemia and necrosis, a multi-finger resection was performed, and she was discharged from the hospital 22 days later. She was hospitalized 98 days after birth because she was not eating. Before admission, she ate 20–30 mL per meal, six times a day. On the day of admission, her total milk volume was 50 mL. She was eating 40 mL per meal every 3–4 h 12 days later and discharged.

**Fig. 2:**
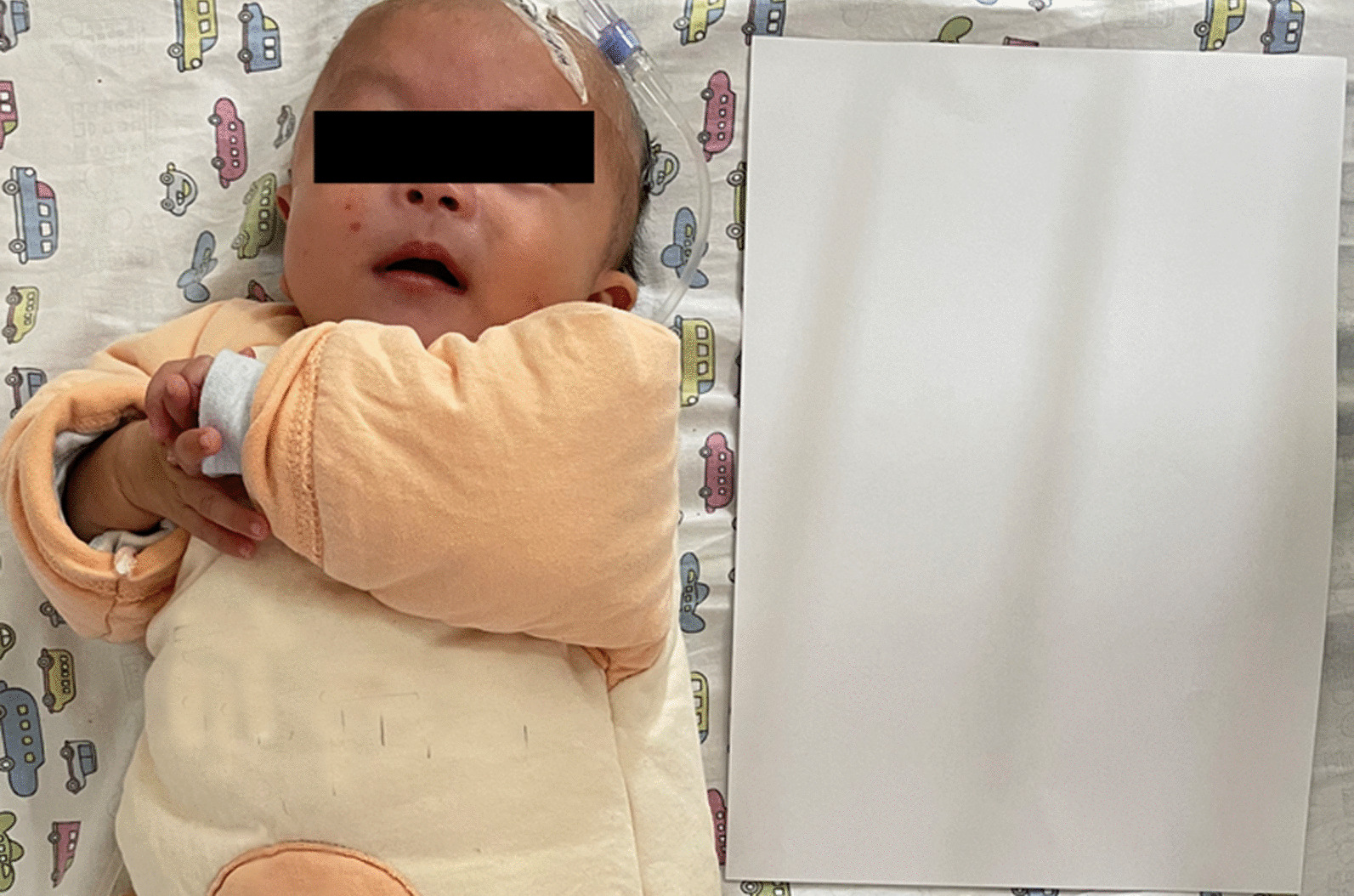
Patient's face and height. Paper is the size of an A4 Sheet

**Fig. 3:**
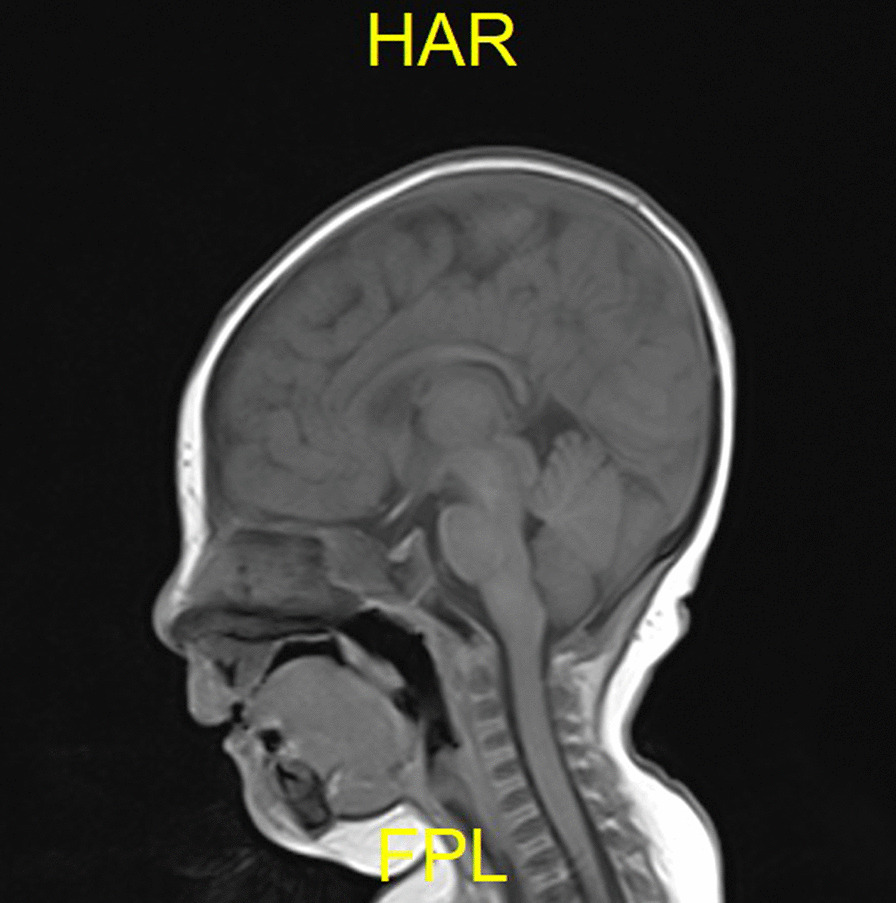
MRI of the child's head

**Fig. 4:**
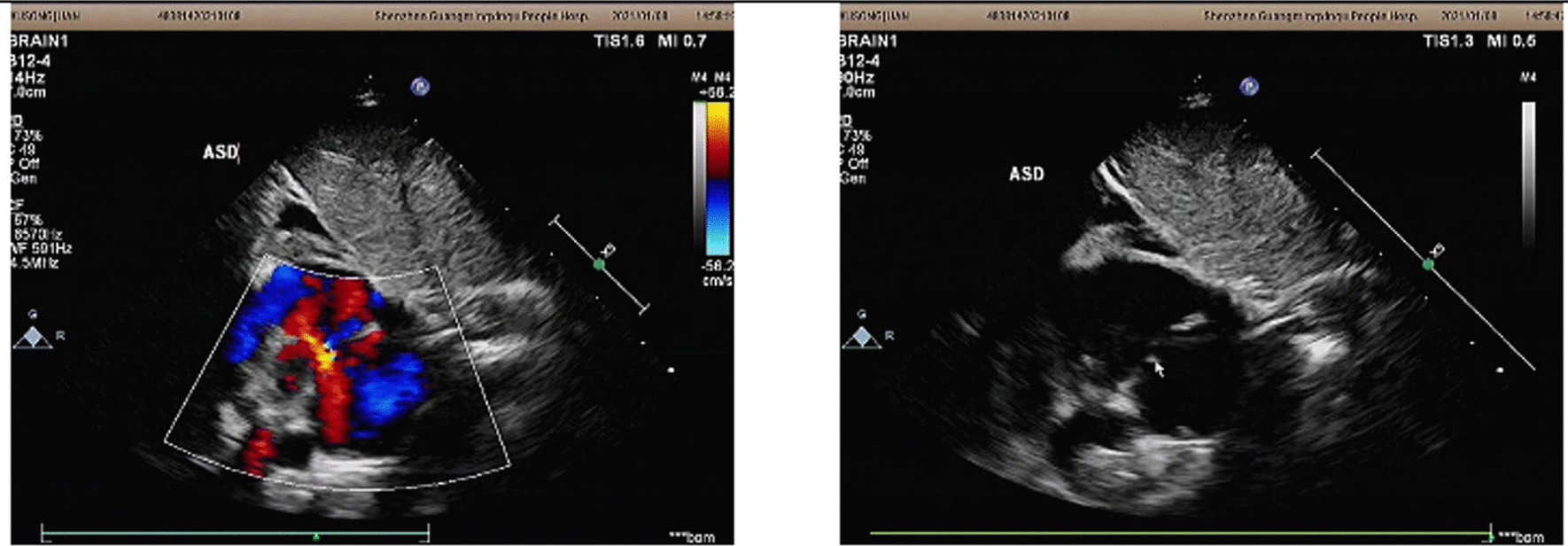
Image of Color Doppler echocardiography

Before the present admission, she ate 50 mL per meal every 3–4 h. Physical examination indicated the following: head circumference 34 cm (unchanged from birth), body temperature 37.8 °C, pulse 146 per min, respirations 42 per min, blood oxygen saturation 95%, weight 4 kg (< 3rd percentile), and body length 53 cm (< 3rd percentile) (Fig. 2). The fontanels were open. Her development was delayed (neck muscle weakness; could stand for 1 min, head height < 3rd percentile, could not support chest and abdomen for one minute on elbows). The pursuit of sounds and objects was normal. She was conscious, had an auricle deformity, warm limbs without cyanosis, and CRT 1.5 s. The patient has no cleft lip/ palate. Her sucking force was inferior, and muscle strength in both arms was slightly reduced, but both legs were normal and she could move her legs freely. The physiological reflexes were present, and no pathological reflexes were elicited. Brain magnetic resonance imaging (MRI) indicated an abnormal signal in the bilateral occipital subarachnoid space, suggestive of leptomeningitis or subarachnoid hemorrhage. Considering the possibility of an abscess, an enhanced MRI was performed. There were no apparent abnormalities in brain ultrasound or MRI of the head (Fig. 3). Color Doppler echocardiography showed a continuous 2.7 mm interruption in the middle of the atrial septum (Fig. 4). Re-examination was recommended after one year of age to rule out a patent foramen ovale. The systolic and diastolic functions of the heart were normal.

**Fig. 5:**
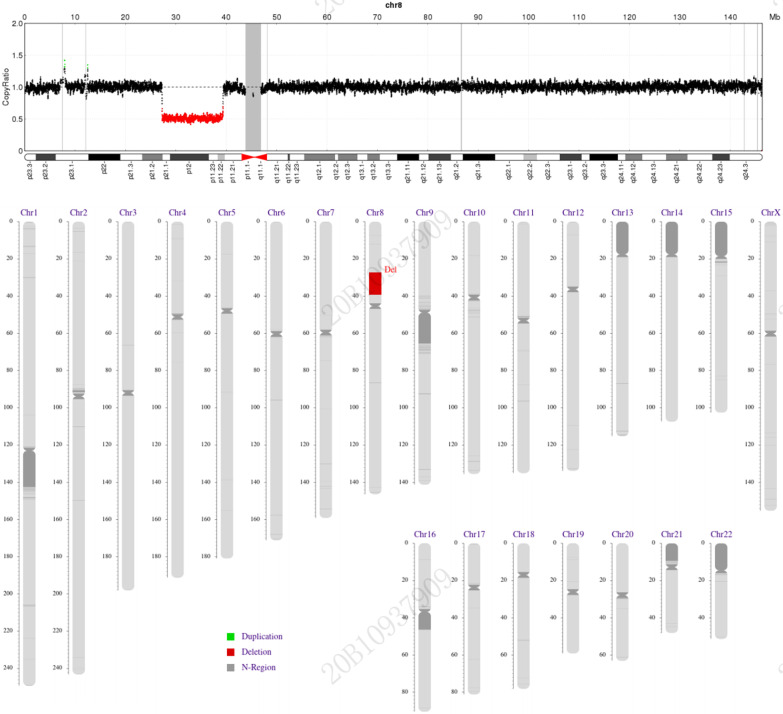
Detection of chromosome defects by high-throughput sequencing

Due to the postnatal growth and psychomotor retardation, auricle deformity, microcephaly, polydactyly, cardiac abnormality, and feeding difficulties, low-depth genome sequencing was performed at Shenzhen Huada Gene Research Institute with the consent of her family (Fig. 5). This showed SEQ (grch37) del (8p21.2p11.22) chr8. A 12 MB pathogenic fragment, G.27228261–39230720del, was from an unknown source. The comprehensive ClinGen CNV score was ≥ 0.99, indicating a pathogenic lesion. The parents' and older brothers’ chromosomes were normal.

As a result of the chromosomal disease, the patient's postnatal growth and development were delayed, and she had muscle weakness, insufficient food intake, and severe pneumonia. As she was weakly positive for *Mycoplasma*, she was given erythromycin and atomization treatment. Her cough improved with rehabilitation and physiotherapy. At discharge, she could hold her head up, remain upright for 2–3 min, and support her chest and abdomen on her elbows for a few minutes. She consumed 50 mL of milk every 3–4 h.

## Discussion

Short arm deletion of chromosome 8 is rare. Since 1975, there have been 15 reports of deletion in 8p11pter (including 6 of deletion in 8p11.1–8p21.1) (Table 1). The common clinical manifestations of chromosome 8 short arm deletion are microcephaly, developmental delay, gonadal hypoplasia, difficulty eating, unique facial features, and spherocytosis. In addition, our patient had six fingers on one hand and external ear malformations. Similarly to this case, only one previously reported case had an abnormal ear [6] due to a chromosome 8p short arm deletion. To the best of our knowledge, it is the first report that the patient with chromosome 8 short arm deletion has polydactyly as one of the clinical manifestations.

**Table 1:** Comparison of present case to previously reported cases of 8p interstitial deletion

Clinical characteristic	1	2	3	4	5	6	7	8	9	10	11	12	13	14	15	Present case
Deletion 8p	p21-p23	p11-p21	p11.1-p21.1		p11.22-p21.1	p11.1-p21.1	p11-p21		p11.2-p21	p21.1-p23.1	p11.23-p21.3	p11.23-p21.1	p12-p22	p11.2	p12-p21.2	p11.22-p21.2
Sex	M	F	F	F	M	M	F	M	M	M	F	M	M	M	F	F
Psychomotor retardation	+	+	+	+	+	+	+	+	NA	+	+	+	+	–	+	+
Growth retardation	+	+	+	+	+	+	NA	NA	NA	+	NA	+	–	+	+	+
Microcephaly	–	+	–	–	+	+	+	+	NA	+	+	+	+	–	+	+
Facial dysmorphism	+	+	+	+	+	+	+	+	+	+	+	+	+	+	+	+
Ocular abnormality	NA	NA	+	+	NA	+	NA	NA	NA	NA	–	+	+	–	+	NA
Hypogonadism	NA	+	NA	NA	+	+	+	+	+	+	NA	+	+	+	+	NA
Neuropathy	NA	NA	+	+	NA	NA	+	NA	NA	NA	NA	+	NA	–	+	NA
Cardiac anomaly	–	–	NA	NA	NA	NA	–	–	NA	+	+	+	–	–	–	+
Spherocytosis	NA	NA	+	+	+	+	+	+	–	NA	–	+	NA	+	–	–

1,Orye and Craen [1976]; 2, Beighle et al. [1977]; 3,4, Chilcote et al. [1987]; 5, Kitatani et al. [1988]; 6, Lux et al. [1990]; 7,8, Cohen et al. [1991]; 9,Stratton et al. [1992]; 10, Marino et al. [1992]; 11, Tsukahara et al. [1995];12, Okamoto et al. [1995]; 13, Devriendt et al. [1999];14, Vermeulen et al. [2002].;15, Klopocki et al. [2006]

This case study describing the deletion of chromosome 8p11.22-p21.2 is the first report of this occurrence, although there are several reported cases of 8p11.1-p21.1 or 8p11.22-p21.1. Kitatani et al. [7] reported an 8p11.22-p21.1 deletion mutation in a 13-month-old boy who presented with postnatal growth and psychomotor retardation, microcephaly, a high arch back, epicanthus, penile cryptorchidism, hypoplasia of two fingernails, sacral depression, and spherocytosis. Okamoto et al. [8] reported a deletion of 8p11.23-p21.1 in a 30-month-old boy who was 48 cm long at birth, with a head circumference of 33.5 cm, stunted development, a heart defect, difficulty feeding, multiple deformities, and spherocytosis. [9] detected an 8p12-p21.2 deletion mutation in a 2.5-year-old girl with developmental delays, facial deformity, and feeding difficulties, but without spherocytosis.

The missing region was found to contain 65 protein-coding genes using whole-genome sequencing, one of which was *FGFR1* (Table 2). The *FGFR1* deletion is associated with Kallmann syndrome [3], hypogonadotropic hypogonadism with anosmia, or hypo-olfaction. In this case, the patient was assisted in smelling milk, vinegar, and soy sauce, respectively, and her facial expression was monitored. From her facial expressions, it was ascertained that she could distinguish different tastes. Other common features of the *FGFR1* deletion include microcephaly, growth retardation, and facial features. The gonadal growth of our patient cannot yet be assessed. However, microcephaly, growth retardation, and facial features are consistent with Kallmann syndrome.

**Table 2:** The deleted genes in this case

Name	Description	Location
ADAM32	ADAM metallopeptidase domain 32	8:39106990-39284917
ADAM9	ADAM metallopeptidase domain 9	8:38996754-39105445
ADGRA2	Adhesion G protein-coupled receptor A2	8:37784191-37844896
ADRB3	Adrenoceptor beta 3	8:37962990-37966599
ASH2L	ASH2 like, histone lysine methyltransferase complex subunit	8:38105493-38144076
BAG4	BAG cochaperone 4	8:38176533-38213301
BRF2	BRF2 RNA polymerase III transcription initiation factor subunit	8:37843268-37849861
CCDC25	Coiled-coil domain containing 25	8:27733316-27772653
CHRNA2	Cholinergic receptor nicotinic alpha 2 subunit	8:27459756-27479883
CLU	Clusterin	8:27596917-27614700
DCTN6	Dynactin subunit 6	8:30156319-30183639
DDHD2	DDHD domain containing 2	8:38225218-38275558
DUSP26	Dual specificity phosphatase 26	8:33591330-33600023
DUSP4	Dual specificity phosphatase 4	8:29333064-29350684
EIF4EBP1	Eukaryotic translation initiation factor 4E binding protein 1	8:38030534-38060365
ELP3	Elongator acetyltransferase complex subunit 3	8:28089673-28191156
EPHX2	Epoxide hydrolase 2	8:27490781-27545564
ERLIN2	ER lipid raft associated 2	8:37736601-37758422
ESCO2	Establishment of sister chromatid cohesion N-acetyltransferase 2	8:27771949-27812640
EXTL3	Exostosin like glycosyltransferase 3	8:28600469-28755599
FBXO16	F-box protein 16	8:28348287-28490278
FGFR1	Fibroblast growth factor receptor 1	8:38400215-38468834
FUT10	Fucosyltransferase 10	8:33370824-33473146
FZD3	Frizzled class receptor 3	8:28494205-28574267
GOT1L1	Glutamic-oxaloacetic transaminase 1 like 1	8:37934281-37940124
GSR	Glutathione-disulfide reductase	8:30678066-30727846
GTF2E2	General transcription factor IIE subunit 2	8:30578318-30658236
HMBOX1	Homeobox containing 1	8:28890395-29064764
HTRA4	HtrA serine peptidase 4	8:38974228-38988663
INTS9	Integrator complex subunit 9	8:28767661-28890242
KCNU1	Potassium calcium-activated channel subfamily U member 1	8:36784324-36936125
KIF13B	Kinesin family member 13B	8:29067278-29263124
LEPROTL1	Leptin receptor overlapping transcript like 1	8:30095408-30177208
LETM2	Leucine zipper and EF-hand containing transmembrane protein 2	8:38386207-38409527
LSM1	LSM1 homolog, mRNA degradation associated	8:38163335-38176730
MAK16	MAK16 homolog	8:33485182-33501262
MBOAT4	Membrane bound O-acyltransferase domain containing 4	8:30131671-30144665
NRG1	Neuregulin 1	8:31639222-32855666
NSD3	Nuclear receptor binding SET domain protein 3	8:38269704-38382272
NUGGC	Nuclear GTPase, germinal center associated	8:28021964-28083936
PBK	PDZ binding kinase	8:27809624-27838082
PLEKHA2	Pleckstrin homology domain containing A2	8:38901235-38973912
PLPBP	Pyridoxal phosphate binding protein	8:37762595-37779768
PLPP5	Phospholipid phosphatase 5	8:38263130-38269243
PNOC	Prepronociceptin	8:28316986-28343355
PPP2CB	Protein phosphatase 2 catalytic subunit beta	8:30774457-30814314
PTK2B	Protein tyrosine kinase 2 beta	8:27311482-27459391
PURG	purine rich element binding protein G	8:30995802-31033715
RAB11FIP1	RAB11 family interacting protein 1	8:37858618-37899497
RBPMS	RNA binding protein, mRNA processing factor	8:30384511-30572256
RNF122	Ring finger protein 122	8:33547754-33567128
SARAF	Store-operated calcium entry associated regulatory factor	8:30063003-30083208
SCARA3	Scavenger receptor class A member 3	8:27633868-27676776
SCARA5	Scavenger receptor class A member 5	8:27869883-27992673
SMIM18	Small integral membrane protein 18	8:30638580-30646064
STAR	Steroidogenic acute regulatory protein	8:38142700-38150992
TACC1	Transforming acidic coiled-coil containing protein 1	8:38728186-38853028
TEX15	Testis expressed 15, meiosis and synapsis associated	8:30831544-30913008
TM2D2	TM2 domain containing 2	8:38988808-38996824
TTI2	TELO2 interacting protein 2	8:33473386-33513185
UBXN8	UBX domain protein 8	8:30729131-30767006
UNC5D	UNC-5 netrin receptor D	8:35235475-35796550
WRN	WRN RecQ like helicase	8:31033788-31176138
ZNF395	Zinc finger protein 395	8:28345590-28402701
ZNF703	Zinc finger protein 703	8:37695782-37700019

The *ANK1* mutation or deletion in the proximal region of 8p11.2 has been linked with spherocytosis [10]. Sequencing revealed no *ANK1* mutation or deletion in this case and no anemia caused by spherocytosis, similar to a case of 8p11.1p21 deletion reported by [11], which had the Kallmann syndrome phenotype but no spherocytosis.

Four genes have been reported among the 65 missing genes: GSR (glutathione disulfide reduction), NRG1 (neuregulin 1), EXTL3 (exostatin-like glycosyltransferase 3), and WRN (WRN RecQ like helicase). In some reports, the complete absence of GSR [12] or homozygous mutations in the gene for GSR [9] are a cause of hemolytic anemia related to low levels of glutathione. Conversely, the level of GSR activity in patients reported by Chilcote et al. [13] and Okamoto et al. [8] was not considered low enough to cause hemolysis through GSR deficiency. The patient in this case study also did not develop hemolytic anemia. NRG1 plays an essential role in the nervous system and heart development [14]. Neurological symptoms such as muscle hypotonia or absent/decreased reflexes were described in four other patients with interstitial 8p deletions [6, 8, 13]. In this case study, her sucking force was inadequate, and the muscle strength of both arms was slightly reduced, but that of both legs was normal. Okamoto et al. [8] described one patient with a deletion containing the EXTL3 gene with retinal dysplasia who was virtually blind. In the current case study, the patient's vision is normal, and there are no other reports correlating the EXTL3 gene with loss of vision. The mutation of WRN is often associated with Werners’ syndrome, an autosomal recessive disorder characterized by the premature onset of a number of age-related diseases [15]. Compared with other reports detailing patients lacking these genes (GSR, NRG1 and EXTL3), the clinical manifestations are different and the mechanisms needs to be closely investigated. In this case, the patient is too young to assess whether Werners syndrome will emerge.

The chromosomes of both parents and older brother were normal, and they displayed no symptoms related to a chromosome deletion. Therefore, the patient's disease is likely due to a de novo mutation in meiosis and needs further investigation.

This case study describes the first patient experiencing the deletion of chromosome 8p11.22-p21.2, affecting 65 protein-coding genes. The limitation of this study is the challenge of confirming whether the specific gene deletion was directly associated with the newly reported phenotypes. The symptoms observed in the female newborn are microcephaly, developmental delays, gonadal hypoplasia, difficulty eating, polydactyly, unique facial features and external ear malformations. On the other hand, due to the discharged young age, some symptoms cannot be fully characterized, such as primary failure of sexual development and visual acuity. Due to this disease's rarity, clinical experience in diagnosing and treating this disease is lacking, with no detailed analysis of bone abnormalities, renal agenesis, and multiple developmental defects.

In conclusion, we report a new 12 MB deletion in the short arm 8p11.22-p21.2 of chromosome 8. Clinically, the prenatal diagnosis should be straightforward due to the many missing fragments on chromosome 8. To date, there is no effective treatment for diseases caused by chromosome deletion, leaving prenatal screening as the primary strategy to prevent genetic defects.

## Data Availability

The raw data have been deposited in the SRA database under the accession number PRJNA719751. The rest of the data that support the conclusions of this study are available from the corresponding author upon request.

## Abbreviations

kg: Kilogram
min: Minute
h: Hour
mL: Milliliter
s: Second
°C: Degrees Celsius
mm: Millimeter

## References

[1] Kimberling WJ, Fulbeck T, Dixon L, Lubs HA (1975). Localization of spherocytosis to chromosome 8 or 12 and report of a family with spherocytosis and a reciprocal translocation. Am J Hum Genet.

[2] White RA, Birkenmeier CS, Lux SE, Barker JE (1990). Ankyrin and the hemolytic anemia mutation, nb, map to mouse chromosome 8: presence of the nb allele is associated with a truncated erythrocyte ankyrin. Proc Natl Acad Sci U S A.

[3] Dodé C, Levilliers J, Dupont JM, De Paepe A, Le Dû N, Soussi-Yanicostas N, Coimbra RS, Delmaghani S, Compain-Nouaille S, Baverel F (2003). Loss-of-function mutations in FGFR1 cause autosomal dominant Kallmann syndrome. Nat Genet.

[4] Mu W, Tochen L, Bertsch C, Singer HS, Barañano KW (2019). Intracranial calcifications and dystonia associated with a novel deletion of chromosome 8p11.2 encompassing SLC20A2 and THAP1. BMJ Case Rep.

[5] Tham E, Lindstrand A, Santani A, Malmgren H, Nesbitt A, Dubbs HA, Zackai EH, Parker MJ, Millan F, Rosenbaum K (2015). Dominant mutations in KAT6A cause intellectual disability with recognizable syndromic features. Am J Hum Genet.

[6] Cohen H, Walker H, Delhanty JD, Lucas SB, Huehns ER (1991). Congenital spherocytosis, B19 parvovirus infection and inherited interstitial deletion of the short arm of chromosome 8. Br J Haematol.

[7] Kitatani M, Chiyo H, Ozaki M, Shike S, Miwa S (1988). Localization of the spherocytosis gene to chromosome segment 8p112.2––8p21. Hum Genet.

[8] Okamoto N, Wada Y, Nakamura Y, Nakayama M, Chiyo H, Murayama K, Inoue T, Kanzaki A, Yawata Y, Hirono A (1995). Hereditary spherocytic anemia with deletion of the short arm of chromosome 8. Am J Med Genet.

[9] Klopocki E, Fiebig B, Robinson P, Tönnies H, Erdogan F, Ropers HH, Mundlos S, Ullmann R (2006). A novel 8 Mb interstitial deletion of chromosome 8p12-p212. Am J Med Genet A.

[10] Eber SW, Gonzalez JM, Lux ML, Scarpa AL, Tse WT, Dornwell M, Herbers J, Kugler W, Ozcan R, Pekrun A (1996). Ankyrin-1 mutations are a major cause of dominant and recessive hereditary spherocytosis. Nat Genet.

[11] Stratton RF, Crudo DF, Varela M, Shapira E (1992). Deletion of the proximal short arm of chromosome 8. Am J Med Genet.

[12] Loos H, Roos D, Weening R, Houwerzijl J (1976). Familial deficiency of glutathione reductase in human blood cells. Blood.

[13] Chilcote RR, Le Beau MM, Dampier C, Pergament E, Verlinsky Y, Mohandas N, Frischer H, Rowley JD (1987). Association of red cell spherocytosis with deletion of the short arm of chromosome 8. Blood.

[14] Falls DL (2003). Neuregulins: functions, forms, and signaling strategies. Exp Cell Res.

[15] Lebel M (2001). Werner syndrome: genetic and molecular basis of a premature aging disorder. Cell Mol Life Sci.

